# Circular RNAs Involve in Immunity of Digestive Cancers From Bench to Bedside: A Review

**DOI:** 10.3389/fimmu.2022.833058

**Published:** 2022-04-04

**Authors:** Chunyue Chen, Congcong Xia, Hao Tang, Yirun Jiang, Shan Wang, Xin Zhang, Tao Huang, Xiaoqing Yuan, Junpu Wang, Li Peng

**Affiliations:** ^1^ Department of Pathology, Xiangya Hospital, Central South University, Changsha, China; ^2^ Xiangya School of Medicine, Central South University, Changsha, China; ^3^ Department of Pathology, School of Basic Medicine, Central South University, Changsha, China; ^4^ Guangdong Provincial Key Laboratory of Malignant Tumour Epigenetics and Gene Regulation, Guangdong-Hong Kong Joint Laboratory for RNA Medicine, Sun Yat-Sen Memorial Hospital, Sun Yat-Sen University, Guangzhou, China; ^5^ Breast Tumour Center, Sun Yat-Sen Memorial Hospital, Sun Yat-Sen University, Guangzhou, China; ^6^ National Clinical Research Center for Geriatric Disorders, Xiangya Hospital, Central South University, Changsha, China; ^7^ Medical Research Center, Sun Yat-Sen Memorial Hospital, Sun Yat-Sen University, Guangzhou, China

**Keywords:** circular RNA, tumor immunity, tumor vaccine, digestive cancers, gastric cancer, colorectal cancer, liver cancer

## Abstract

The immune system plays a complex role in tumor formation and development. On the one hand, immune surveillance can inhibit the growth of tumors; on the other hand, immune evasion of tumors can create conditions conducive for tumor development and growth. CircRNAs are endogenous non-coding RNAs with a covalently closed loop structure that are abundantly expressed in eukaryotic organisms. They are characterized by stable structure, rich diversity, and high evolutionary conservation. In particular, circRNAs play a vital role in the occurrence, development, and treatment of tumors through their unique functions. Recently, the incidence and mortality of digestive cancers, especially those of gastric cancer, colorectal cancer, and liver cancer, have remained high. However, the functions of circRNAs in digestive cancers immunity are less known. The relationship between circRNAs and digestive tumor immunity is systematically discussed in our paper for the first time. CircRNA can influence the immune microenvironment of gastrointestinal tumors to promote their occurrence and development by acting as a miRNA molecular sponge, interacting with proteins, and regulating selective splicing. The circRNA vaccine even provides a new idea for tumor immunotherapy. Future studies should be focused on the location, transportation, and degradation mechanisms of circRNA in living cells and the relationship between circRNA and tumor immunity. This paper provides a new idea for the diagnosis and treatment of gastrointestinal tumors.

## 1 Background

In 1976, Sanger et al. (1) discovered a new single-stranded covalent circular RNA (circRNA) in plant viroids that is different from linear RNA. Subsequently, they proposed the concept of circRNA. CircRNA has been long regarded as a by-product of incorrect alternative splicing (2) and thus has not attracted the attention of the academic community. With the advancement in sequencing and bioinformatics technologies, our knowledge of circRNAs significantly increased. CircRNA is an endogenous non-coding RNA (ncRNA) molecule widely found in various biological cells. The most significant difference between circRNA and linear RNA is that the 3′ end and 5′ end of the circRNA molecule are connected to form a closed-loop structure. This particular structure provides circRNA with a high degree of abundance, conservation, stability, and distinctive expression pattern, which is tissue-specific and related to the developmental stage (3). At present, there are six main functions of circRNAs: (i) as a sponge of microRNAs (miRNAs), (ii) co-acting with proteins, (iii) regulation of gene transcription, (iv) selective splicing regulation, (v) as a translation template for proteins, and (vi) regulation of tumor immunity (3–6). More in-depth knowledge of circRNA can provide a new research direction to explore the occurrence, development mechanism, treatment, and prognosis of many diseases.

Digestive cancers mainly include oesophageal cancer, gastric cancer (GC), colorectal cancer (CRC), liver cancer, gallbladder cancer, cholangiocarcinoma, and pancreatic cancer. According to the Global Cancer Statistics 2020, the cancers with high mortality in 185 countries worldwide are lung cancer, liver cancer (8.3% of the cancer-related deaths), GC (7.7%), breast cancer, CRC (9.2%), oesophageal cancer (5.5%), pancreatic cancer (4.7%), and gallbladder cancer (0.9%) (7), with digestive cancers accounting for leading cancer deaths (36.3%). Similarly, the incidence of digestive cancers is estimated to reach as high as 5,091,327 new cases by 2040, which accounts for 26.4% of all 36 cancer types (7). These data indicate that cancers of the digestive system pose a serious health burden worldwide.

In the digestive system, immune tissue is widely distributed. The liver, digestive tract lymph node and mucosal immune tissue all perform different immune functions. The immune response plays a crucial role in the invasion of endogenous or exogenous substances. The role of immunity in tumors is similar, which is closely related to the occurrence and development of tumors (1–4). Immune surveillance and immune escape lead to different outcomes. Furthermore, circRNAs can regulate the expression of host genes. More principally, they serve as microRNA (miRNA) sponges to adjust the axis of miRNA/mRNA or regulate the activities of immune cells directly. Thus they play an essential role in the immune disorders of tumors (5–8). This paper summarizes the relationship between circRNA and digestive tumors, mainly focusing on tumor immunity.

## 2 Biosynthesis of circRNA

In general, eukaryotic genes remove non-coding intron sequences through pre-mRNA splicing after transcription. And the exon sequences containing protein-coding information are sequentially joined together to form mature linear RNA molecules, thus achieving the transmission of genetic information. However, there is a method of reverse splicing, i.e., the reverse head-to-tail connection of the gene exon sequence, that forms circRNAs. The vast majority of circRNAs in organisms are processed and synthesized by precursor RNA (pre-mRNA) through various nonclassical reverse splicing methods.

**Figure 1:**
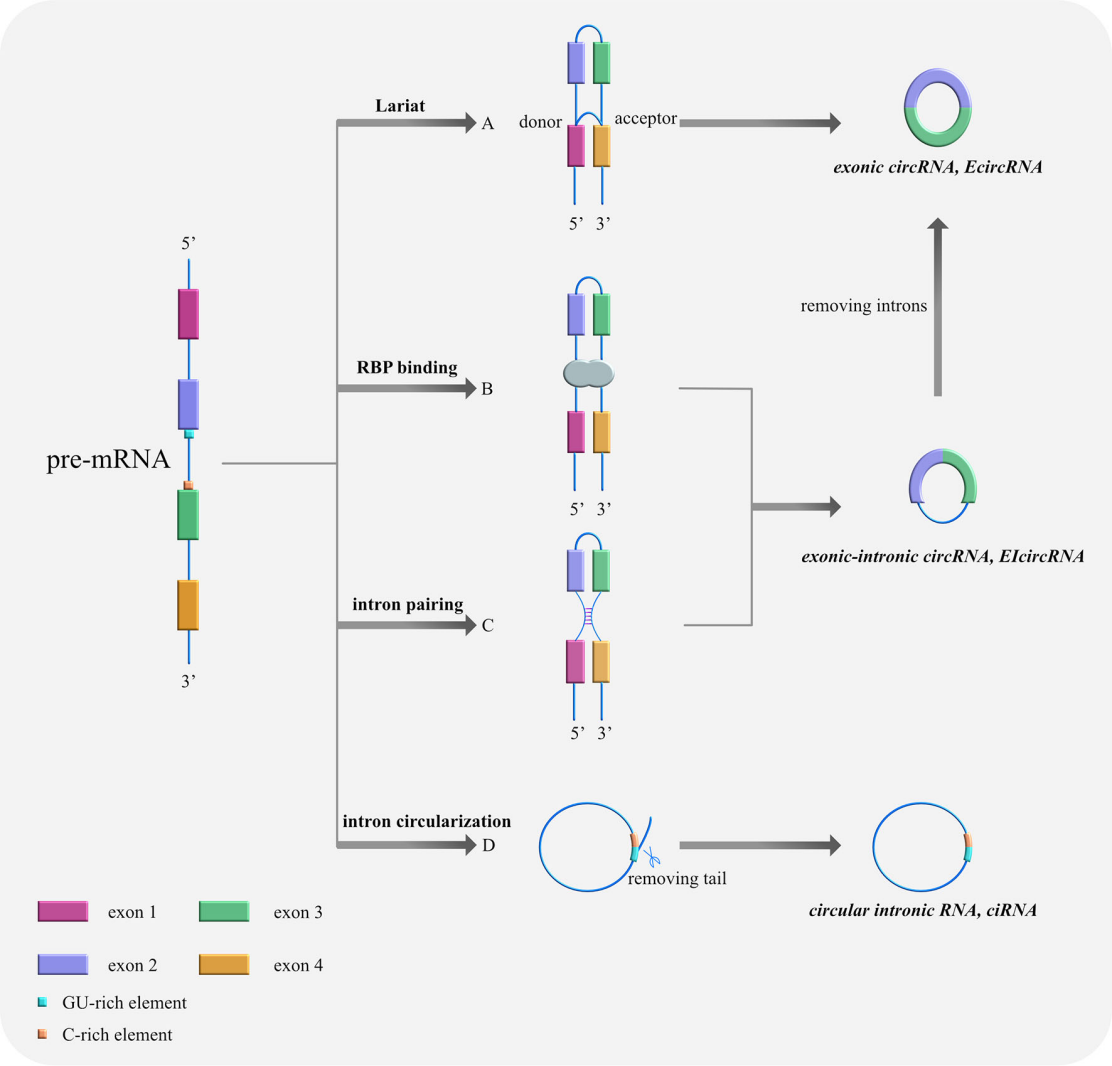
Cyclization models of circRNAs. **(A)** Lariat-driven cyclization. The 5′ donor splice site downstream of exon 4 is connected with the 3′ receptor splice site upstream of exon 1, forming a reverse covalently linked lariat structure. Then, the lasso containing skipped exons shears further and removes the introns, thus forming ecircRNA. **(B)** RNA-binding protein (RBP)-driven cyclization. RBPs bind to the splicing sites of both sides of exons 2 and 3 to assist cyclization and form ElciRNA. **(C)** Cyclization driven by intron pairing: complementary sequences at the flanks of exons 2 and 3 bring splicing sites together by base pairing, forming double-stranded RNA side by side. Afterwards, ecircRNA containing exons or EIciRNA containing exons and introns are produced through variable shearing. **(D)** Self-cyclization of introns: Pre-mRNA is spliced by spliceosome to remove intervening introns and there are only exons left, which are connected to form mature RNA. With the 7nt GU-rich sequence at both ends of the intron, the U-rich sequence, and the 11nt C-rich sequence, an exon-free lariat RNA structure is formed by 2′,5′ connection’s circularization.

Based on their composition, circRNAs can be divided into three categories as follows (**Table 1**): (i) exonic circRNAs (ecircRNAs), which are composed entirely of exons (9), (ii) exonic-intronic circRNAs (EIcircRNAs), which contain both exons and introns (10), and (iii) circular intronic RNAs (ciRNAs), which are composed entirely of introns (11). The splicing formation of circRNA can be classified into two categories: exon-cyclized and intron-cyclized. Jeck et al. (9) proposed three different formation models for ecircRNAs and EIcircRNAs. First is the Lariat-driven cyclization in which the 5′ donor site downstream of one exon is connected to the 3′ receptor site upstream of another exon to form a reverse covalently linked lasso structure. That is, the lasso-driven splicing donor and the splice acceptor are covalently combined to form an annular structure. After further splicing and removing the introns, the exons form ecircRNA through phosphodiester bonds. At this time, the synthesized circRNA is mainly located in the cytoplasm (12) (**Figure 1A**). The second model is RNA-binding protein (RBP)-dependent cyclization (13). Here, the upstream and downstream introns at both ends of the pre-mRNA exon bind specifically to RBP. Then, through a sequence that RBP can recognize, a dimer forms. Finally, the dimer cycles to form EIciRNA. At this time, the synthesized circRNA is mainly located in the nucleus (**Figure 1B**). The third model is cyclization driven by intron pairing. The introns on both sides of the pre-mRNA exons contain reverse complementary sequences, which form double-stranded RNA side by side at the splicing site in the duplex. Finally, ecircRNA containing exons or EIciRNA containing exons and introns are formed through differential splicing (14). Here, the synthesized circRNA is mainly located in the nucleus (**Figure 1C**). The ciRNA formed by the self-cyclization of introns is different from that of EcircRNA. It contains a unique 2′,5′ connection whose formation is related to the 7nt guanine and uracil-rich (GU-rich) sequence at both ends of the intron, the uracil-rich (U-rich) sequence, and the 11nt cytosine-rich (C-rich) sequence. The two ends of the intron GU-rich and C-rich elements are cyclized to form an exon-free lasso RNA structure with different tail lengths (15). After removing the tail branches, ciRNA is generated mainly in the nucleus (11) (**Figure 1D**).

**Table 1:** Similarities and differences among the three types of circRNA.

Characteristics	EcircRNA	EIcircRNA	ciRNA
Formation mode	Exon cyclization	Exon cyclization	Intron cyclization
Formation pattern	Lariat-driven cyclization	RBP-dependent cyclization or cyclization driven by intron pairing	Driven by GU-rich and C-rich components
Constitution	Exon	Exon and intron	Intron
Location	Cytoplasm	Nucleus	Nucleus
Parental gene location	Intragenic	Intragenic	Intragenic

Based on the gene location of the parents, circRNAs can be classified as intragenic and intergenic circRNAs. Each ecircRNA, EIcircRNA, and ciRNA molecule is derived from the splicing of different exons and/or introns within the same parent gene, so they are all considered intragenic circRNAs. Meanwhile, circRNA from the genome interval between different genes is called the intergenic circRNA.

## 3 Properties of circRNA

CircRNAs are produced by special alternative splicing and largely exist in the cytoplasm of eukaryotic cells (2), although a small number of intron-derived circRNAs are found within the nucleus (16). Besides, circRNAs have solid specificity of cell, tissue, and development (17, 18). They are also widely found in human cells. In fact, more than 10% of the genes can produce circRNAs through reverse splicing and other pathways. The circRNA of some genes accumulates in the cells, whose number far exceeds that of linear mRNAs corresponding to the gene, and sometimes even up to ten times that of their linear isomers (19, 20). Furthermore, unlike the traditional linear RNA with 5′ and 3′ ends, the circRNA molecule has a covalently closed loop structure without a 5′- terminal cap and a 3′- terminal poly (A) tail; thus, it is not easily degraded by exonuclease RNase R and is more stable than linear RNA (19, 21, 22). Besides, many circRNAs are evolutionarily conserved among different species (19, 20). Jeck et al. (9) discovered that 457 of 2121 murine circles that readily mapped from human genomes could produce murine circular RNA, at a rate of 22%. The homology of mouse and human circRNAs varies from less than 5% to nearly 30% (23), whereas that of pig and mouse circRNAs varies from less than 15% to nearly 20% (24), and that of mouse and rat circRNAs varies ≥23% (25). In addition, some circRNAs exhibit rapid evolutionary changes (5, 16). Most circRNAs are derived from exons, and a small portion is formed by the direct circularisation of introns. Moreover, circRNA molecules are rich in miRNA response elements (MREs), which can act as miRNA sponges and competitive endogenous RNA (ceRNA) binding to miRNA. In this way, circRNAs can relieve the inhibition of miRNA on its target genes and up-regulate the expression level of target genes (14). Most circRNAs are non-coding RNAs (20). Jeck et al. (19) confirmed that there are more than 25 000 different RNAs in human fibroblasts that could produce circRNA. In addition, the same gene can produce different circRNAs through variable cyclization (16, 20); thus, circRNAs from the same gene source are diverse and enrich the species of circRNA. Other features of circRNAs remain to be elucidated. Compared with traditional linear RNA, circRNA has more quantity, higher stability and more MRE. It has great advantages in research, making it more likely to be used in the mechanism exploration and treatment of gastrointestinal tumors.

## 4 circRNA and Tumor Immunity

Studies have shown that circRNA is differentially expressed in cancer compared with normal tissues, which suggests that they serve a specific function in these cells (19). And exo-circRNA may distinguish patients with cancer from healthy controls, which illustrates its significant translational potential as a circulating biomarker for cancer diagnosis (26). These indirectly prove the role of circRNA in tumor development, and tumor immunity is an important factor determining the direction of tumor development. Therefore, the relationship between circRNA and tumor immunity is worth exploring.

To date, an increasing number of studies have confirmed that circRNAs play a critical role in immune response and the regulation of tumor immunity. For example, several circRNAs are associated with the infiltration of immune cells into tumors. Du et al. have reported that B and T cells infiltrate the tumor and the surrounding connective tissue in breast cancer and express circ-Foxo3. It indicates a host immune response to tumor xenografts, with circ-Foxo3 playing a tumor-suppressive role (27). Zou et al. also showed that circ-CDR1as is crucial for infiltrating immune cells in tumor tissues, especially CD8+ T cells (28). Furthermore, a ceRNA network involves hsa-miR-494, circ-UBAP2, and five central genes, particularly *ZEB1* and *CXCR4*. The network can regulate pancreatic adenocarcinoma progression by controlling the infiltration of immune cells (16). CircRNA is also involved in tumor immunity by regulating intercellular cell adhesion molecule-1 (ICAM-1) molecules. In response to tumor cell invasion, hsa_circ_0007456 can regulate ICAM-1 expression *via* miR-6852-3p, thereby regulating natural killer (NK) cell activity to mediate tumor immune escape (17). Interestingly, in viral immunity, another circRasGEF1B molecule plays the opposite role. CircRasGEF1B regulates the level of ICAM-1 by modulating the stability of its mRNA, thus stimulating the innate immune response and protecting host cells from pathogens (18).

CircRNAs in tumors are also closely linked to immune-related molecules. For example, the nuclear factor kappa B (NF-κB) has been shown to play a unique role in the immunity of tumor cells (20–22). Its inhibition attenuates the ciRS-7-induced upregulation of MMP-2, thereby inhibiting ciRS-7-mediated oesophageal squamous cell carcinoma cell invasion (23). Wang et al. revealed that the circRNA-000911/miR-449a/Notch1/NF-κB network might be partly responsible for the oncogenic activity of breast cancer cells and maybe a new target for breast cancer therapy (24).

### 4.1 Relationship Between the Basic Functions of circRNA and Tumor Immunity

**Figure 2:**
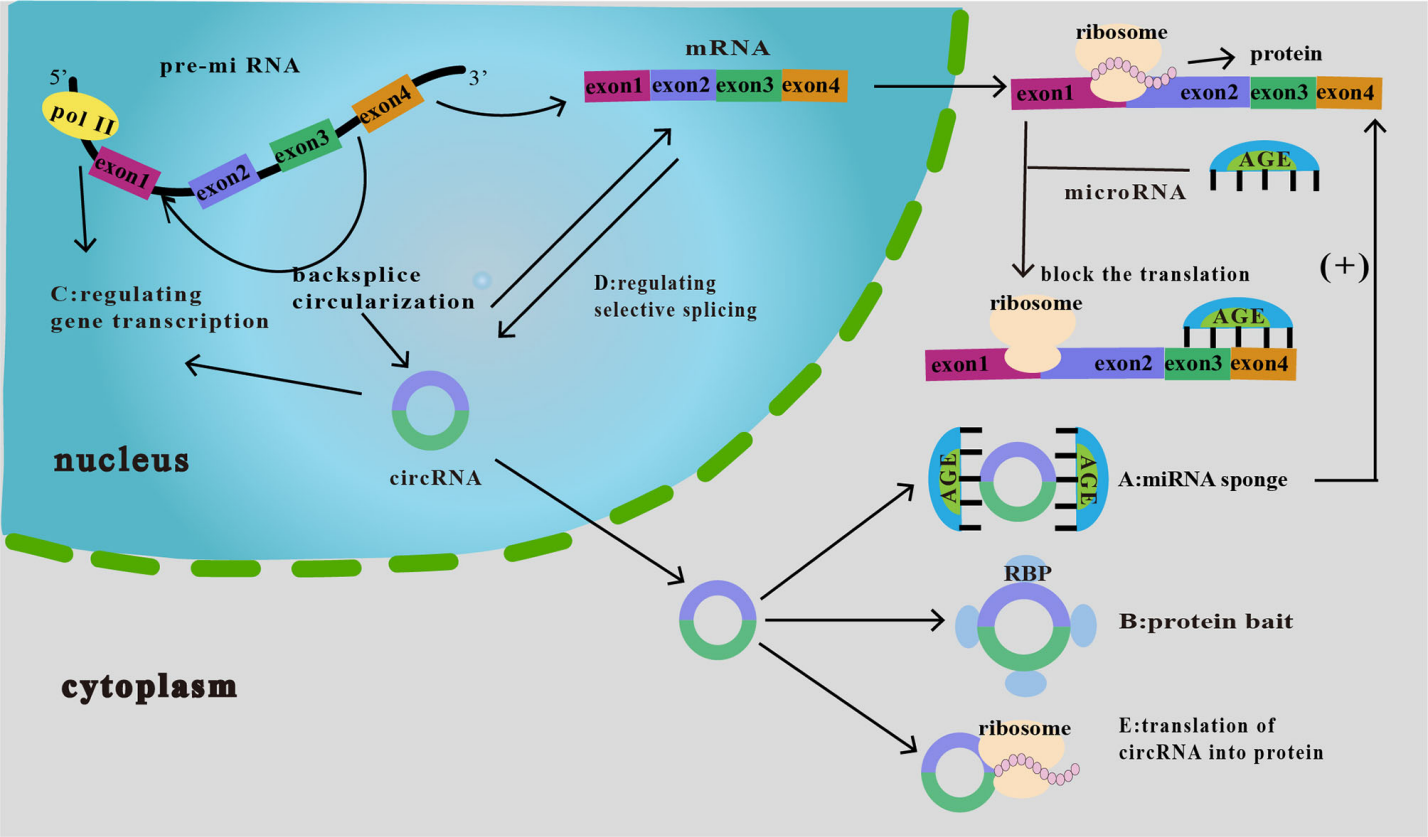
Functions of circRNAs. **(A)** As miRNA sponge. CircRNA binds miRNAs and inhibits their downstream responses, which then increases the expression of downstream target proteins, thereby affecting tumor proliferation, invasion, and migration. **(B)** As protein bait. CircRNAs act as protein decoys or antagonists to inhibit protein function, thus affecting the related progression of cancer. **(C)** Regulation of gene transcription. CircRNA interacts with RNA polymerase II to regulate the transcription and expression of parental genes. **(D)** Regulation of selective splicing. CircRNAs compete with linear RNAs; if the formation of circRNAs increases, linear splicing decreases. **(E)** Translation of circRNA into protein. CircRNAs can be translated into proteins in an unusual cap-independent manner.

The primary known functions of circRNA include the following: (i) acts as a molecular sponge for miRNAs to silence the corresponding miRNAs and cause a series of alterations in downstream responses (25) (**Figure 2A**); (ii) interacts with a protein (protein decoy), thereby inhibiting its function (**Figure 2B**); (iii) regulation of gene transcription (**Figure 2C**); (iv) regulation of selective splicing (**Figure 2D**); and (v) translation into proteins (**Figure 2E**). Although current studies on the relationship between circRNAs and tumor immunity have mainly focused on their role as miRNA molecular sponges and protein decoys, studies on the regulation of selective splicing have also been conducted. Therefore, these three functions will be the main focus of our discussion.

#### 4.1.1 As a miRNA Sponge

MiRNAs are endogenous non-coding RNAs with regulatory functions in eukaryotic organisms and are roughly 19–25 nucleotides in length. They are involved in the formation of RNA-induced silencing complexes (RISC) and direct RISC to regulate post-transcriptional silencing (29, 30). However, circRNAs contain MREs that bind to miRNAs and act as miRNA sponges or ceRNAs, thus silencing the corresponding miRNAs and causing a series of alterations in downstream responses (25). Many of the genes with known ceRNA interactors identified so far have been implicated in human disease. Aberrant changes in ceRNA regulation may also contribute to disease initiation and progression (31). So ceRNA network interactions may have a role in determining the effectiveness of RNA-directed therapies (32). As a kind of Cerna, the role of circRNA in disease development is also worth studying.

**Figure 3:**
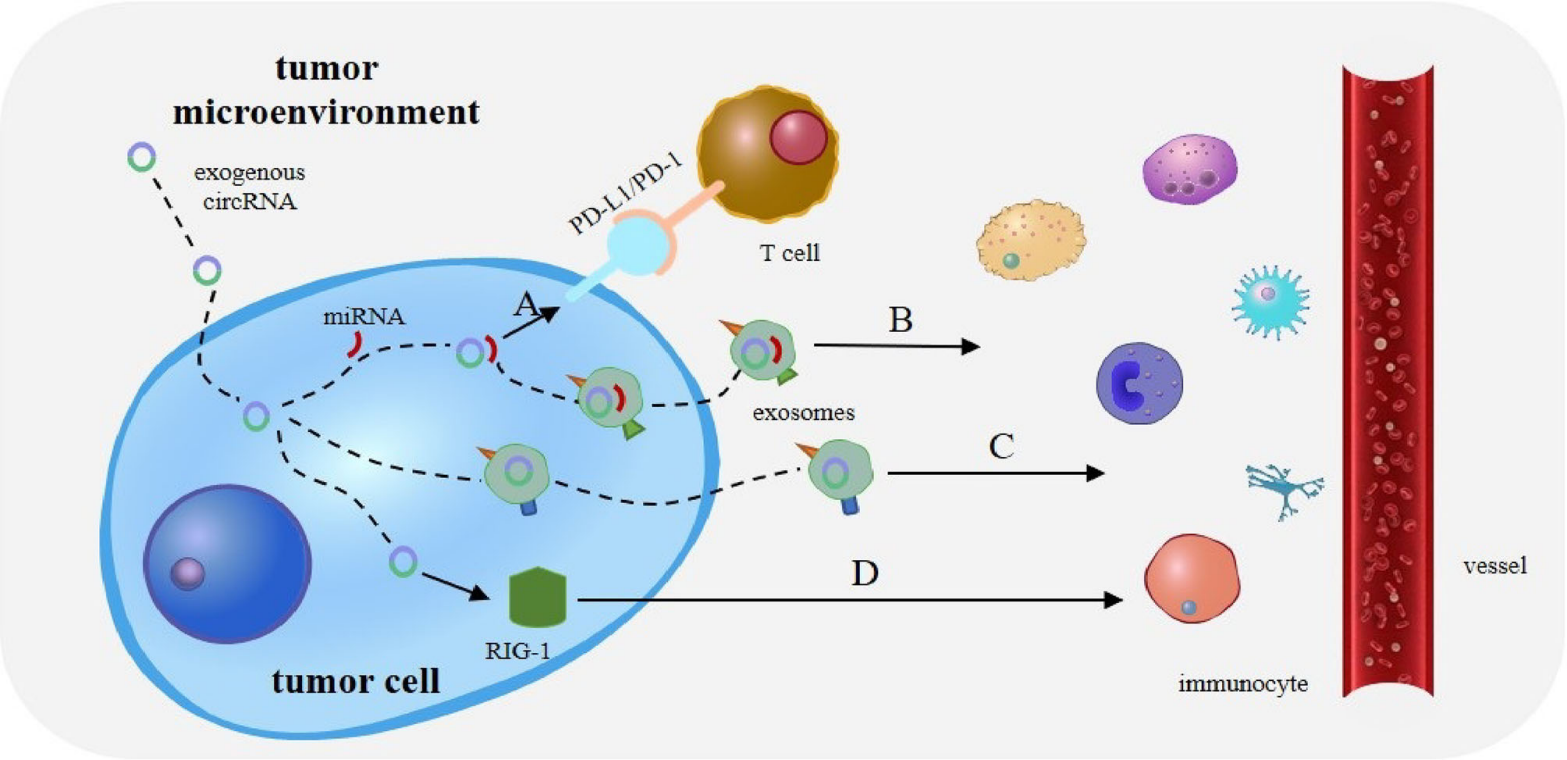
Role of circRNAs in tumor immunity. **(A)** The interaction between programmed death receptor 1 (PD-1) and programmed cell death ligand 1 (PD-L1) can effectively suppress the activation of effector T lymphocytes, ultimately leading to tumor immune escape. **(B)** CircRNA can also bind tumor-specific miRNAs and mRNAs through complementary sequences, to form a novel tumor antigen. CircRNA exists in exosomes to enhance the stability of these RNAs and is released upon reaching the target immune cells. **(C)** Exosomes and extracellular vesicles (EVs) can transport circRNA into immune cells. CircRNA can then act as potential tumor antigens to regulate immune responses in tumors. **(D)** Exogenous circRNA entering tumor cells may activate antitumor immunity by influencing RIG-I.

CircRNAs regulate immunity in various types of cancer by acting as a miRNA sponge, and much attention has been paid to the regulation of immune escape. The interaction between programmed death receptor 1 (PD-1) and programmed cell death ligand 1 (PD-L1) can effectively suppress the activation of effector T lymphocytes, ultimately leading to tumor immune escape (**Figure 3A**). CircRNA-002178 has also been found to bind to miR-34a and suppress its expression, resulting in increased PD-L1 expression in cancer cells. CircRNA-002178 can also be transported from cancer cells to CD8+ T cells *via* exosomes. It suppresses miR-28-5p activity and promotes PD1 expression on CD8+ T cells (33). In addition, miR-138 activity is inhibited upon binding to hsa_circ_0020397, thus affecting its downstream responses and promoting the expression of PD-L1 and telomerase reverse transcriptase in CRC cells. Owing to the high expression level of has_circ_0020397 in CRC cells, the expression of PD-L1 is upregulated in turn (34, 35). Similarly, circRNA vimentin (circ-VIM) can bind to miR-124 and deregulate its downstream target PD-L1. Sevoflurane can synergistically inhibit immune escape from oesophageal cancer by circ-VIM silencing (36). The role of miRNAs in tumor immunity, especially in the regulation of PD-1/PD-L1 immune checkpoint expression, has been extensively investigated (37, 38). These results suggest that circRNAs promote tumor immune escape through the circRNA/miRNA/PD-1/PD-L1 axis.

In breast cancer, circ_0000977 binds to miR-153 to counteract the inhibition of hypoxia-inducible factor 1-α (HIF1A) and a disintegrin and metalloproteinase domain-containing 10 (ADAM10) (39). However, hypoxia levels decrease the level of NKG2D on NK cells by inducing the upregulation of HIF1A, ADAM10, and soluble MICA, consequently tumor cells evade immune surveillance and cell lysis (40, 41). Thus, the circ_0000977/miR-153 axis may regulate the immune escape of breast cancer cells by increasing the levels of HIF1A and ADAM10 (39). In addition, circ-Amotl1 can indirectly inhibit the presence of miR-17-5p by upregulating DNMT3A. The process may lead to DNA methylation enrichment in the promoter region of miR-17 and result in subcellular translocation and STAT3 expression, thereby initiating immunosuppression in tumors (42, 43). These studies suggest that circRNAs can regulate the function of miRNAs related to cancer immunity. Further exploration of the relationship between circRNAs and miRNAs is needed to provide new ideas for tumor immunotherapy.

Interestingly, the binding of circRNA to miRNA has other effects besides miRNA silencing. CircRNA can also bind tumor-specific miRNAs and mRNAs through complementary sequences to form a novel tumor antigen. CircRNA also exists in exosomes to enhance the stability of these RNAs and is released upon reaching the target immune cells (35) (**Figure 3B**). This suggests that the role of circRNA as a miRNA sponge in tumor immunity needs to be explored further.

#### 4.1.2 As Protein Decoys

CircRNA can also act as a protein decoy or antagonist. CircRNA may serve as a decoy and bind to an intracellular protein to inhibit its function. Many endogenous circRNAs tend to form 16–26 bp double-stranded bodies (dsRNA) and inhibit the activity of the endogenous protein kinase (PKR) (44). In the model proposed by Zheng, this repression was achieved by the interaction between circRNA and PKR (45). However, after viral infection, endogenous circRNA can be degraded by RNase L to regulate dsRNA production, activate PKR, and promote innate immunity (44). In addition, circFoxo3 binds to p53-associated murine double minute2 to induce p53 degradation. P53 plays a crucial role in tumor development by increasing inducible nitric oxide synthase expression and regulating the immune response (46, 47). It is suggested that circRNA could regulate the immune response in tumors by regulating the stability of p53.

In non-small cell lung cancer (NSCLC), circNDUFB2 also inhibits NSCLC progression by disrupting insulin-like growth factor 2 mRNA-binding proteins and activating anti-tumor immunity. The inhibition can also be enhanced by N6-methyladenosine (m6A) modification of circNDUFB2. Therefore, circNDUFB2 may be involved in NSCLC progression as a potential tumor suppressor (48). However, not all circRNAs that bind to a protein can inhibit protein function. For instance, ectopic circ-Amotl1 binds to the nuclear oncogene c-myc and stabilizes it, thereby upregulating c-myc targets and promoting tumorigenesis (49).

#### 4.1.3 Regulation of Selective Splicing

Previously, we mentioned that circrna activates retinoic acid-induced gene I (RIG-I) and protects against viral infections through the immune pathway. In particular, circRNAs activate RIG-I *via* self-splicing introns, rather than the same circRNAs produced by endogenous introns and spliced by a cellular splicing mechanism. Thus, the activation of this immune response depends on the splicing mechanism of circRNAs (18). Furthermore, in preclinical models of hematological malignancies, the linear form of human plasmacytoma variant translocation 1 (PVT1) is overexpressed in myeloid-derived suppressor cells. The overexpression could induce immune tolerance. However, circPVT1 exhibits immunosuppressive properties in myeloid and lymphocyte subpopulations (50). Linear pvt1 and circpvt1 play two different roles in the immune regulation of hematological malignancies. The former involves cancer progression by promoting a more aggressive phenotype of malignant cells, and the latter presents a new potential therapy resistance mechanism to negatively regulate the immune system (50). This study provides new insights into the treatment of hematological malignancies and demonstrates the unique immunomodulatory role of circRNA.

### 4.2 Regulation of Antitumor Immunity as Tumor Antigens

Owing to their stability and specificity, circRNAs can act as tumor antigens and induce immune responses. Exosomes and extracellular vesicles (EVs) can transport circRNA to immune cells (51), thus acting as potential tumor antigens to regulate immune responses in tumors (**Figure 3C**). For example, CRC cells with KRAS mutations can transfer several circRNAs into exosomes (52). Many abnormal circRNAs are produced during tumorigenesis owing to gene mutations and chromosomal changes. In acute promyelocytic leukaemia, the chromosomal translocation of PML/RARα produces fused circRNAs (53). Newly formed abnormal circRNAs may then be transported to immune cells through exosomes and EVs secreted by tumor cells (35).

In addition, exogenous circRNAs can trigger immune responses in HeLa cells and stimulate the expression of innate immune genes, thus preventing viral infection (54), which may be achieved through their immunogenicity. Many circRNAs that regulate immune responses by inducing the expression of immune genes have been identified, including RIG-I (55, 56) and MDA5 (44, 54). RIG-I is a critical factor in the host antiviral defense system (57). It can isolate viral RNA from antiviral effector molecules, thus triggering an antiviral immune response (18). Subsequently, it can help the body better detect exogenous circRNA and form a positive feedback pathway, which can protect the body against viral infection (54). In addition, RIG-I agonists can activate anti-cancer immune responses against tumors (58, 59).

Exogenous circRNAs entering tumor cells may activate antitumor immunity by influencing RIG-I (**Figure 3D**). For example, circNDUFB2 can be recognized by RIG-I to activate the RIG-I MAVS signal cascade and recruit immune cells into the tumor microenvironment (TME) (48). Interestingly, exogenous circRNA without modification can avoid cellular RNA sensors. So it can avoid triggering an immune response in RIG-I and toll-like receptor cells and mice, resulting in reduced immunogenicity (60). In lung cancer, the overexpression of cirNDUFB2 leads to the overregulation of various immune genes and increased levels of CXCL10, CXCL11, CCL5, and IFNβ, thus enhancing the body’s immune defense (48).

Recently, the relationship between m6A modification and circRNA immunity has attracted much attention. Studies have shown that human circRNAs are labelled at birth by covalent m6A modification based on introns that program reverse splicing. The m6A modifier is marked as “SELF”, whereas the unmodified is marked as “FOREIGN”. RIG-I differentiates the unmodified and the m6A-modified circRNAs and is activated only by the former (61). CircSELF can evade innate immune surveillance through YTHDF2 (m6A reader protein)-mediated inhibition (62), which may inhibit RIG-I conformational conversion (activation) required for downstream signaling of immune genes in living cells (61).

Consistent with this, m6A-modified RNAs can be raised by the YTHDF protein and induced into disordered condensates of phase separation through its N-terminal domain (63). Furthermore, the human papillomavirus, which is closely related to the growth of CaSki cervical cancer cells *in vitro* and *in vivo*, can produce the oncogenic circRNA CircE7. In addition, m6A modification was found to be an essential motif of CircE7 protein-coding ability (64).

All in all, these studies show that the modification of m6A helps exogenous circRNAs evade immune surveillance. CircFOREIGN is also involved in immune signal transduction by forming a three-component signal transduction complex with RIG-I and K63-polyubiquitin chain (K63-UBN) (61).

Overall, circRNAs are involved in some critical processes of host immunity by maintaining internal homeostasis and providing adequate protection against microbial infection and malignancy. Thus, circRNA can be considered as a biomarker or therapeutic target. However, the underlying mechanisms remain largely unknown and should be further explored and clarified.

## 5 Role of circRNA in Digestive Cancer Immunity

Although progress has been made in the treatment of digestive tract tumors, the tumor immune microenvironment limits the effect of therapy to a certain extent. As mentioned above, the incidence and mortality of digestive cancers remain high: CRC ranks 5th and 2nd, GC ranks 6th and 3rd, and liver cancer ranks 7th and 5th in new morbidities and cancer mortalities, respectively (65). Tumor immunity-related studies have focused on these three types of tumors. Therefore, we mainly discuss them in this review.

Immune tissue is widely distributed in the digestive system. The liver and digestive tract lymph nodes perform different immune functions. In addition, there is a mucosal immune system in the digestive tract, mainly gut-associated lymphatic tissue (GALT). The mucosa of the gastrointestinal tract can perform its innate immune function through recruitment of neutrophils and killing effect of IL-23/Th17 cell axis. In addition, the intestinal epithelium can produce protective sIgA due to the activation of adaptive immunity. Immune tolerance also exists: dendritic cells (DCs) recognize gut microbes, regulatory T cells are activated and begin to secrete interleukin-10 (IL-10) (66).

For the liver, it always maintains a low immune response state to avoid some unnecessary immune responses. But when it comes to tumor cells or other situations, the liver exerts its immune effect. The balance of immune tolerance and immune protection is important to maintain normal liver immune function. However, if immune tolerance is dominant, it may cause tumor metastasis and progression. In the liver microenvironment, Kupffer cells (KCs) show poor activation of adaptive immune response which leads to immune tolerance rather than immune protection (67, 68). And when NK cell function is impaired, it can result in limited tumor cell clearance. In addition, the innate immune cells, such as dendritic cells, innate lymphocytes cells, are all involved in the immune response of the liver (69).

During tumor development, tumor cells secrete cytokines, inflammatory mediators, and chemokines, leading to the reprogramming of the surrounding cells. The TME comprises tumor cells, stromal cells, immune cells, endothelial cells, and non-cellular components, playing an essential role in immune regulation (70). On one hand, tumor cells exhibit antigenic components that are different from normal cell tissues. Presenting tumor antigens to T cells can generate cellular immunity, humoral immunity, and cellular cytotoxic reactions of innate immune cells. These immune processes act as a defence in the early stages (71, 72). On the other hand, tumor cells can evade immune cells through complex signaling networks to achieve immune escape (70, 73). CircRNAs participate in many pathological processes, including cancer growth, metastasis, recurrence, and treatment resistance (74–80). These processes are realized primarily by sponging miRNA and interacting with proteins.

In the tumor immune microenvironment, circRNAs achieve immune escape through the following aspects:

- Regulation of NK cell activity.
- Regulation of macrophage activity. In response to different stimuli, macrophages differentiate into M1 or M2 macrophages to perform their functions. CircRNAs may affect macrophage secretion.
- Regulation of lymphocyte activity. The proportion of tumor-infiltrating lymphocytes (TILs) in the TME is high and is closely related to prognosis. The regulation of circRNA on T lymphocyte PD-1 can improve tumor immune tolerance.
- Interaction with other immune-related factors (51, 73, 81).

We will introduce the role of circRNAs in the immunity of GC, CRC, and liver cancer in detail below (**Table 2**). The functions above are equally applicable to digestive cancers.

**Table 2:** CircRNA involved in the immune regulation of various types of digestive cancers.

Immunocyte	CircRNA	Type of digestive cancer	Immune surveil-lance	Immune escape	Molecular mechanism	References
Natural killer cells	circARSP91	Liver cancer	+		Regulation of ULBP1 expression	(82)
	circUHRF1 (hsa_circ_0048677)	Liver cancer		+	miR-449c-5p/TIM-3	(83)
	circTRIM33-12	Liver cancer		+	miR-191/TET1/NKG2D	(84)
	hsa_circ_0007456	Liver cancer		+	miR-6852-3p/ICAM-1	(17)
Macrophages	hsa_circ_0074854	Liver cancer		+	differentiation into M2 macrophages, secretion of IL-10	(85)
	has-circ-0110102	Liver cancer		+	miR-580-5p/PPARα/CCL2 influence COX-2/PGE2	(86)
	circASAP1	Liver cancer		+	miR-326 or miR-532-5p/CSF-1	(87)
	circTMC5	Gastric cancer		+	miR-361-3p/RABL6	(88–90)
Lymphocytes	circMET (CD8 +T lymphocyte)	Liver cancer		+	miR-30-5p/Snail/DPP4/CXCL10	(91)
	circ-0020397 (T lymphocyte)	Colorectal cancer		+	miR-138/PD-L1/PD-1	(34)
	CircCDR1-AS (T lymphocyte)	Colorectal cancer		+	CMTM4, CMTM6/PD-L1/PD-1	(92)
	hsa_circ_0136666 (T lymphocyte)	Colorectal cancer		+	miR-497/PD-L1/PD-1	(93)
	circ-KRT6C (T lymphocyte)	Colorectal cancer		+	miR-485-3p/PD-L1/PD-1	(94)
	circTMC5 (CD8+T lymphocyte, B lymphocyte)	Gastric cancer		+	miR-361-3p/RABL6	(88–90)
	has_circ_0064428 (TILs)	Liver cancer		+	——	(95)
Dendritic cells	circTMC5	Gastric cancer		+	miR-361-3p/RABL6	(88–90)
Neutrophile granulocytes	circTMC5	Gastric cancer		+	miR-361-3p/RABL6	(88–90)

+ indicates the role of CircRNA in immune surveillance or immune escape in a certain gastrointestinal tumor.

### 5.1 Immunity of GC

Although circRNAs have been reported to show many potential functions in tumorigenesis and tumor immune reactions, these functions are far from fully understood. As the relationship between miRNAs and tumor immunity is more evident than that between circRNAs presently, the role of circRNA in anti-tumor immunity is mainly understood through the circRNA/miRNA/mRNA axis. Peng et al. reported that circTMC5 targets RABL6 in GC by acting as a sponge for miR-361-3p to regulate the biological phenotype of GC cells (88). In addition, RABL6 participates in the immune regulation of GC through the production of IL-6 and IL-8, activation and transport of leukocytes, promotion of neuroinflammatory responses, and activation of macrophages during inflammation (89). The TISIDB database revealed the correlation between RABL6 and chemokines, including chemokine CCL14, CCL22, and CXCL3, in human GC samples using Spearman correlation (90). Among them, IL-6 and IL-8 can promote the genesis and development of tumors (96, 97), and CXCL3 may promote the malignant biological behavior of tumors (98, 99). In addition, a negative correlation between RABL6 expression and the infiltration of immune cells, including macrophages, B cells, CD8^+^T cells, neutrophils, and dendritic cells, has been observed (90). Thus, combining checkpoint blockade therapy with therapies that target this pathway may be a potential treatment strategy (100). Overall, the circTMC5/miR-361-3p/RABL6 axis plays a crucial role in the occurrence and development of GC and affects the tumor immune microenvironment.

The Epstein–Barr virus (EBV) is the earliest human tumor-associated virus (101). The EBV-associated gastric carcinoma is a group of GC in which EBV infects gastric epithelial cells during monoclonal growth (102, 103). One of its characteristics is the immune microenvironment in which viruses or virus-infected cells can grow normally. EBV BARTs are produced in large numbers in epithelial cells with EBV latent infection, and circRNAs have been identified in the BART loci; however, the function of circBARTs remains unclear. In addition, circRNA can be detected in the serum or plasma of patients because of its stability. EBV-specific circRNAs have great potential for future applications in this field.

### 5.2 Immunity of CRC

#### 5.2.1 Immune Surveillance

As previously described, circRNA can be transported into immune cells by exosomes and EVs and act as potential tumor antigens to modulate immune reactions in tumors (51). For example, circRNA is downregulated and transferred by exosomes to KRAS-mutated colon cancer cells (52), demonstrating that it may act as a tumor antigen.

#### 5.2.2 Immune Evasion

CircRNA can promote the immune escape of CRC cells through PD-L1. In CRC cells, circRNA CIRC-0020397 binds to miR-138 and inhibits the activity of miR-138, thereby facilitating downstream reactions, such as the production of telomerase reverse transcriptase and PD-L1. Since the expression of CIRC-0020397 is elevated in CRC cells, PD-L1 is upregulated. CIRC-0020397 interacts with PD-1 to induce T cell apoptosis, which causes immune escape. Studies have shown that PD-1/PD-L1 blockers provide better clinical benefits in patients with high PD-L1 levels (34). Similar studies have also reported that cirCCDR1-AS can significantly increase PD-L1 expression on the surface of colon cancer cells through CMTM4 and CMTM6, leading to a poor prognosis (92). Hsa_circ_0136666 promotes PD-L1 expression in CRC by inhibiting miR-497, which stimulates the production of Treg cells and causes immune escape (93). Circ-KRT6C can improve the presentation of PD-L1 by acting as a miR-485-3p sponge, thus promoting the malignant progression and immune escape of CRC cells (94). Therefore, targeting the relevant circRNAs to regulate PD-1/PD-L1 expression may provide a new direction for immune checkpoint therapy.

In addition, recent studies have proposed that the exosome CIRC-ABCC1 can bind to β-catenin to enter the nucleus and activate the Wnt pathway, which promotes the progression of CRC. Thus, circRNAs can promote malignant biological behavior and assist in the diagnosis and treatment of CRC.

### 5.3 Immunity of Liver Cancers

#### 5.3.1 Immune Surveillance

The NKG2D ligand ULBP1 is upregulated by circARSP91 at mRNA and protein levels, thereby enhancing the responsiveness of hepatocellular carcinoma (HCC) cells to NK cells (82). However, the miRNA sponge does not achieve the above effect, which has been speculated to be related to tumor suppressors (104).

#### 5.3.2 Immune Escape

##### 5.3.2.1 Regulation of NK Cell Activity

Compared with normal tissues, the level of circUHRF1 (hsa_circ_0048677) in HCC tissues increases. CircUHRF1 can enter NK cells *via* exosomes to achieve immune escape. Under the action of the circUHRF1/miR-449C-5P/T cell immunoglobulin domain and mucin domain protein-3 axis, NK cell infiltration in TME, and IFN-γ and TNF-α secretion decrease, leading to tumor immune escape. In addition, the above reactions regarding the circUHRF1 can increase HCC resistance to PD-1 therapy and the difficulty of treatment (83). The expression of circTRIM33-12 increases in HCC, leading to the inhibition of miR-191, in which the expression of TET1 is upregulated. Experiments showed a positive correlation between the expression of circTRIM33-12 and the number of NKG2D-positive cells. The decrease in NKG2D level is associated with the immune responses of NK cells, γδT cells, and CD8+ T cells to cancers (84, 105). In addition, hsa_circ_0007456 regulates NK cell activity *via* the hsa_circ_0007456/miR-6852-3p/ICAM-1 axis, which leads to immune escape (17).

##### 5.3.2.2 Regulation of Macrophage Activity

Macrophages with different phenotypes can affect the progression of liver cancer. For example, hsa_circ_0074854 is upregulated in HCC cells, and its knockdown can inhibit the polarization of tumor-associated macrophages (TAM) to M2 (85). As M2 macrophages can contribute to the migration and invasion of tumor cells by secreting IL-10 (106, 107), the knockdown of hsa_circ_0074854 suppresses tumor growth. CircRNA regulation of macrophage infiltration and secretion is also involved in immune escape. The decreased expression of hsa-circ-0110102 suppresses that of PPARα *via* the action of miR-580-5p, thereby affecting the secretion of CCL2. CCL2 can inhibit the release of pro-inflammatory cytokines by regulating the COX-2/PGE2 axis to achieve immune escape (86). CircASAP1 regulates the infiltration of TAM by regulating the miR-326 or the miR-532-5p/CSF-1 pathway, thus affecting the anti-tumor ability of the TME (87).

##### 5.3.2.3 Regulation of Lymphocyte Activity

The overexpression of circMET in HCC induces a tumor immunosuppressive microenvironment through the miR-30-5p/Snail/dipeptidyl peptidase 4/CXCL10 axis. The reduction in CXCL10 level leads to the decreased capacity for CD8 + lymphocyte transport, immune tolerance, and less sensitivity to PD-1 therapy (91). Studies have confirmed that hsa_circ_0064428 level is reduced in HCC patients with high TIL infiltration, and there is a negative relationship between hsa_circ_0064428 level and tumor size, metastasis rate, and survival rate. It is speculated that circRNA can regulate the tumor immune microenvironment by regulating TILs, and hsa-circ-0064428 can be used as a prognostic marker (95).

Blocking the tumor escape pathway associated with circRNA provides new ideas for future tumor therapy. In addition, circRNAs can be used as molecular markers for the diagnosis and prognosis of digestive cancers (108–111). The characteristics of circRNA make it more advantageous over other non-coding RNA. For example, the universality of circRNA make the therapeutic audiences wider and increase the sensitivity of tumor markers; diversity and richness provide the circRNA pathway with more alternative targets.

## 6 Immunotherapy of Digestive Cancers

There are various treatment methods for gastrointestinal tumors, including surgery, endoscopic resection, radiotherapy and chemotherapy, immunotherapy, targeted therapy, and multiple combination therapies. Recently, the success of cancer immunotherapies, such as monoclonal antibodies, immune checkpoint inhibitors PD-1 and CTLA-4, chimeric antigen receptor T-cell, and other therapies, has attracted increasing attention (112). These therapies enhance the anti-tumor ability of the TME by activating or enhancing the body’s immune system, thus killing tumor cells.

Because of the obvious anti-tumor effect, blocking PD-1/PD-L1 pathway has a bright prospect in clinical tumor treatment. The interaction between PD-1 and PD-L1 transmits inhibitory signals to T cells, ultimately leading to a series of tumor immune escape reactions (113, 114). Recent studies have shown that this signaling pathway can block the G1 phase of the T cell cycle and induce iTregs to inhibit T cell activity (115, 116). Various types of circRNA can regulate the PD-1/PD-L1 pathway of digestive tumors through different signaling pathways. Therefore, future studies should investigate the regulation of the PD-1/PD-L1 pathway using circRNAs, which can contribute new ideas to immune checkpoint therapy for digestive tumors.

Cancer vaccines are similar to vaccines for common infectious diseases, including antigens and adjuvants. Adjuvants activate antigen-presenting cells and regulate surface molecules to initiate an adaptive immune response (117). Compared with vaccines for common infectious diseases, cancer vaccines are administered primarily for treatment rather than prevention. The application of circRNAs in tumor vaccines has recently entered the preliminary stage of exploration. Animal experiments showed that circFOREIGN decreased the tumor growth rate and improved the overall survival rate in OVA-B16 melanoma mouse model (61), demonstrating the potential of circRNA as an adjuvant for tumor vaccines. CircRNA can activate dendritic cells to promote antigenic cross-reactivity and activate CD4+ and CD8+T cells. CircRNA may also directly affect T cells and other immune cell types; however, this remains to be explored. Interestingly, the m6A modification of circRNA is recognized by the immune system as body tissue, thus weakening the immunogenicity of circRNA as an adjuvant (61).

To date, mRNA has been shown to be useful in developing cancer vaccines. Compared with traditional mRNA vaccines, the circRNA vaccine has a more stable circRNA ring structure (118); therefore, only a small amount of circRNA is required to initiate adaptive immunity. The CircRNA vaccine is highly expected due to its reduced dosage and improved efficiency. However, the immunogenicity of unmodified circRNA is lower than that of unmodified mRNA *in vitro* and depends on the purity of circRNA (60). Therefore, despite the promising prospects of the circRNA vaccine, its application as a tumor vaccine against gastrointestinal tumors remains challenging.

## 7 Future Perspective

At present, there is no practical way to treat digestive tumors, except surgery, chemotherapy, and radiotherapy. Although significant progress has been made recently in immunotherapy, tumor cells still can lead to immune escape in various ways. That’s because the immunogenicity of tumor antigens is weak. Besides, some tumors have innate or acquired drug resistance, which hinders the progress of immunotherapy. Therefore, it is of great significance to explore the immune mechanisms of digestive tumors to improve the effect of immunotherapy.

Several studies have demonstrated the close relationship between circRNAs and tumor biological processes, such as proliferation, metastasis, and drug resistance. CircRNA can influence the immune microenvironment of gastrointestinal tumors by acting as a miRNA molecular sponge, interacting with proteins, and regulating selective splicing. In addition, by regulating the activities of NK cells, macrophages, lymphocytes, and other immune cells, circRNAs can lead to tumor immune escape. The circRNA vaccine, though just entering the stage of preliminary exploration at present, provides a new idea for tumor immunotherapy.

Since nowadays knowledge of circRNA remains insufficient, future studies should consider focusing on the following issues: (i) so far, most studies on circRNAs and tumors have been carried out on tumor cells and tissues. Studies on other cancer models, such as organoids and patient-derived xenografts, should be conducted; (ii) there are relatively a few circRNAs available, and the available loci are limited; (iii) the downstream reaction of circRNA is not well understood, so it is necessary to explore whether there are other effects; and (iv) as immunotherapy is expected to become more individualized, how to achieve this goal using circRNA will be a complex issue. Future studies should clarify the location, transport, and degradation mechanisms of circRNA in living cells, as well as the relationship between circRNA and tumor immunity. Safety studies should also be carried out to explore new paths for gastrointestinal tumor therapy.

## 8 Conclusions

CircRNAs have become a research hotspot worldwide. In this review, we summarized the relationship between circRNA and digestive tumors. Also, we found out the potential of circRNA as a vaccine against digestive tumors. With the progress of sequencing technology and bioinformatics analysis, knowledge of circRNA and gastrointestinal tumors is expected to be more in-depth. We believe that circRNA will provide a brand-new direction on immunotherapy, which will inevitably become an essential link in tumor therapy in the future.

## Author Contributions

JW, LP, and XY conceived and revised this manuscript. CC, CX, HT, and YJ wrote the manuscript. SW, XZ, and TH helped to draft the manuscript. All authors reviewed and approved the final manuscript.

## Funding

This work was partially supported by the National Natural Science Foundation of China (Project No. 81602167 to JW, Project No. 81803636 to XY, Project No. 81972658 and 81802812 to LP), Hunan Provincial Natural Science Foundation of China (Project No. 2017JJ3494 and 2021JJ31100 to JW), the Science and Technology Program Foundation of Changsha City (Project No. kq2004085 to JW), and Guangdong Basic and Applied Basic Research Foundation (Project No. 2018A0303130329 to XY).

## Conflict of Interest

The authors declare that the research was conducted in the absence of any commercial or financial relationships that could be construed as a potential conflict of interest.

## Publisher’s Note

All claims expressed in this article are solely those of the authors and do not necessarily represent those of their affiliated organizations, or those of the publisher, the editors and the reviewers. Any product that may be evaluated in this article, or claim that may be made by its manufacturer, is not guaranteed or endorsed by the publisher.

## Abbreviations

ADAM10, a disintegrin and metalloproteinase domain-containing 10; ceRNA, competitive endogenous RNA; circRNAs, circular RNAs; ciRNAs, circular intronic RNAs; C-rich, cytosine-rich; CRC, colorectal cancer; dsRNA, double-stranded bodies; ecircRNAs, exonic circular RNAs; EIcircRNAs, exonic-intronic circular RNAs; EVs, extracellular vesicles; GC, gastric cancer; GU-rich, guanine and uracil rich; HIF1A, hypoxia-inducible factor 1-α; ICAM-1, intercellular cell adhesion molecule-1; MREs, miRNA response elements; NF-κB, nuclear factor kappa B; NSCLC, non-small cell lung cancer; PD-1, programmed death receptor 1; PD-L1, programmed cell death ligand 1; PKR, protein kinase; RBP, RNA-binding protein; RIG-I, retinoic acid-induced gene I; RISC, RNA-induced silencing complexes; TILs, tumor-infiltrating lymphocytes; TME, tumor microenvironment; U-rich, uracil-rich.
